# A386G polymorphism and infertility

**DOI:** 10.4103/0974-1208.69338

**Published:** 2010

**Authors:** Viroj Wiwanitkit

**Affiliations:** Department of Laboratory Medicine, Faculty of Medicine, Chulalongkorn University, Bangkok - 10330, Thailand

Sir,

I read the recent publication on A386G polymorphism and infertility by Singh *et al*. with a great interest.[1] Singh *et al*. reached the conclusion that “as in the report from Italy and South India, our results illustrate the rarity of this mutation. Apparently, this mutation is of recent origin and/or has poor selective value.”[1]

Indeed, the clinical correlation between A386G polymorphism of the *DAZL* gene and idiopathic male infertility is still a myth. The difference in the report from different countries might be due to many possible reasons. First, the race and ethnic effect might be a probable factor contributing to different clinical correlation. This needs further systematic assessment for clarification. Second, the polymorphism, a genetic phenomenon, might not be detectable in a few studied subjects. A larger study might be needed for the verification of the findings. Third, there might be no direct correlation between studied polymorphism and male fertility in reality. A new bioinformatics technique might be useful for the clarification of the clinical correlation of A386G polymorphism as used for other polymorphisms.[2]
